# Quantitative size-resolved characterization of mRNA nanoparticles by in-line coupling of asymmetrical-flow field-flow fractionation with small angle X-ray scattering

**DOI:** 10.1038/s41598-023-42274-z

**Published:** 2023-09-22

**Authors:** Melissa A. Graewert, Christoph Wilhelmy, Tijana Bacic, Jens Schumacher, Clement Blanchet, Florian Meier, Roland Drexel, Roland Welz, Bastian Kolb, Kim Bartels, Thomas Nawroth, Thorsten Klein, Dmitri Svergun, Peter Langguth, Heinrich Haas

**Affiliations:** 1 European Molecular Biology Laboratory, Hamburg Unit, Hamburg, Germany; 2 Department of Biopharmaceutics and Pharmaceutical Technology, Johannes Gutenberg-University, Mainz, Germany; 3 BioNTech SE, Mainz, Germany; 4 Postnova Analytics GmbH, Landsberg am Lech, Germany; 5 BIOSAXS GmbH, Hamburg, Germany

**Keywords:** Nanoscale biophysics, Nanomedicine, Techniques and instrumentation, Nanoscience and technology, Nanoparticles

## Abstract

We present a generically applicable approach to determine an extensive set of size-dependent critical quality attributes inside nanoparticulate pharmaceutical products. By coupling asymmetrical-flow field-flow fractionation (AF4) measurements directly in-line with solution small angle X-ray scattering (SAXS), vital information such as (i) quantitative, absolute size distribution profiles, (ii) drug loading, (iii) size-dependent internal structures, and (iv) quantitative information on free drug is obtained. Here the validity of the method was demonstrated by characterizing complex mRNA-based lipid nanoparticle products. The approach is particularly applicable to particles in the size range of 100 nm and below, which is highly relevant for pharmaceutical products—both biologics and nanoparticles. The method can be applied as well in other fields, including structural biology and environmental sciences.

## Introduction

The success of messenger RNA (mRNA) nanoparticles for vaccination against Covid-19 has highlighted the potential of RNA nanomedicines as well as of nano-scaled pharmaceutical products in general^1–5^. In mRNA vaccines, so-called lipid nanoparticles (LNPs), which are characterized by a specific lipid composition and manufacturing process, are used for mRNA delivery. There is a wealth of different other nano-scaled pharmaceuticals which have reached various stages of clinical and preclinical development. The particles may be based on organic (e.g., lipids, polymers, polypeptides, proteins) as well as on inorganic materials (e.g., metals, metal oxides, silica)^6,7^.

The majority of these products are intended for parenteral application with particle sizes typically below 200 nm (the limit for sterile filtration): LNPs measure 100 nm or less, and certain particle formats (e.g., for targeting tumors or crossing the blood–brain barrier) are in the range of tens of nanometers. With dimensions of a few or few tens of Angstroms, other types of drug formats such as biologics (therapeutic proteins, antibodies) or soluble polymers may be included for this category of nano-sized drugs. The characteristics of all these systems are dominated by their colloidal nature, where particle size and size-related attributes are of fundamental importance for quality, biological efficacy, and safety.

Determination of size-related parameters, such as internal structure and especially drug loading, poses particular challenges regarding quality control^8^. Obtaining information on size distribution profiles and size-dependent parameters is crucial for the identification of critical quality attributes (CQA) and critical process parameters (CPP)^9^. Size-related properties are especially important in the development of generic versions of originator products, as equivalence in physicochemical and biological characteristics needs to be demonstrated. In the case of protein-based therapeutics, controlling aggregation and potential denaturation is crucial, while for nanoparticle products size, particle size distribution, and the above-mentioned size-dependent attributes are significant.

Dynamic light scattering (DLS) is a widely accepted technique for regular size measurements in the control of pharmaceutical nanoparticle products. This high throughput method is easily applicable, robust, sensitive to delicate changes of size characteristics, and fulfills many requirements for regular application in quality control laboratories. By using standard algorithms such as the Koppel algorithm^10^, DLS can provide numerical values that represent a length dimension (sphere-equivalent hydrodynamic diameter), interpreted as an average size, as well as a dimensionless value, the polydispersity index, which is used to indicate physical polydispersity. However, these values hold physical meaning primarily for relatively monodisperse samples. In fact, most nanoparticulate pharmaceutical systems, including liposomes, and lipid- or polymer-based nanoparticles, are characterized by a certain degree of intrinsic polydispersity. Quantitative information on the physical size distribution profiles and size-dependent parameters (e.g. loading, release, structure) are of superior importance for the development and quality control of such products^8,9^. There are algorithms that allow to calculate physical profiles from DLS data, but these are highly model-dependent and are usually not suitable for regular use^11^. Other methods that provide at least semi-quantitative insight into size distributions comprise nanoparticle tracking analysis, analytical ultracentrifugation as well as chromatography-based methods such as size exclusion chromatography (SEC) and asymmetrical-flow field-flow fractionation (AF4) in combination with multiple downstream detection systems. However, the individual methods have all certain specific drawbacks and can provide only semi-quantitative profiles, which depend in a complex manner on parameters such as size-dependent scattering intensities, refractive index gradients, densities, molecular conformation, swelling characteristics, and others^11^.

To our best knowledge, so far, no method for in situ determination of absolute size distribution profiles for colloidal particulates in the size range of 100 nm and below is available. Here, we present a novel approach to obtain such data, providing, direct, quantitative size distribution profiles and further quality-indicating parameters by combining AF4 with small angle X-ray scattering (SAXS) measurements.

AF4 is an elution-based fractionation technique, allowing the separation of particulates according to hydrodynamic size in the range of approximately 1–1000 nm. Multiple detectors, including, for example, refractive index, UV absorption, circular dichroism, fluorescence, DLS, and multi angle light scattering (MALS) can be connected in-line to the AF4 separation set-up. For investigation of pharmaceutical nanoparticles, including liposomes and other colloidal systems, and protein-based drug products, it is a frequently applied tool in development^12–17^.

Solution SAXS is a method that provides direct structural information in a size range from hundreds of nanometers down to the sub-Ångstrom scale, depending on instrumental settings. The SAXS signal is directly correlated to the analyte concentration (through the electron density gradient) in a linear manner, therefore it is suitable for absolute concentration measurements^18^. The obtained structural data are model-independent, as directly correlated to the squared autocorrelation function of the electron density distribution, from which real space structure can be revealed by different approaches using standard software packages from the respective research facilities^19^.

SAXS may be considered as one of the ‘gold standard’ methods for the characterization of colloids, nanoparticles, proteins, and polymers in solution, as well as for various biomembrane systems. It is as well established for the characterization of different types of nano-scaled pharmaceutical products^20–24^. Due to the relatively low contrasts (e.g., the electron density gradients between particulates and bulk phase) of soft matter systems, for measurements with laboratory X-ray sources a certain analyte concentration is required. Therefore, for diluted systems with low contrast, measurements are often performed at synchrotrons.

One prerequisite for unambiguous quantitative SAXS data analysis is, that only one type of particulate is present, in an as far as possible monodisperse form. Therefore, for protein samples, for instance, careful sample preparation is important to avoid aggregates which scattering signal would contribute to the scattering curves^25^. A solution routinely adopted to overcome this limitation is to combine SAXS with with a method to separate the analyte as a function of size.

The combination of size-dispersive methods with SAXS has been successfully utilized in the field of structural biology, particularly by directly connecting SEC to the measuring cell, commonly referred to as SEC-SAXS. Here, the benefits of ‘polishing the sample on-the-fly’ was first demonstrated in 2004 at the biological SAXS beamline BioCAT in Chicago (APS, USA)^26^ and have manifested themselves in such a way, that currently over 13 dedicated scattering beamlines (X-ray and neutrons) have implemented this separation technique at their beamlines and are in high demand by the whole global user community^20,21,27^.

However, the use of SEC is not applicable to all systems. Its limitations include for example undesired shear forces through interactions with the stationary phase and restricted resolution in regards to more broadly dispersed samples^28^. In contrast to that, AF4 allows the variation of several instrumental parameters which facilitates the development of tailored methods for a given type of sample or product. AF4 allows separation over a bigger size range and polydispersity level, also for shear-force sensitive samples.

So far, to our knowledge, only one approach for combining AF4 directly with SAXS has been reported, in which polyelectrolytes in aqueous solutions were analysed^29^. The hesitancy for the direct hyphenation of AF4 and SAXS may be attributed to the fact that AF4 as a separation technique is still not as established in routine purification and analytical processes as chromatographic approaches are. In general, its potential is often underestimated^28^.

**Figure 1:**
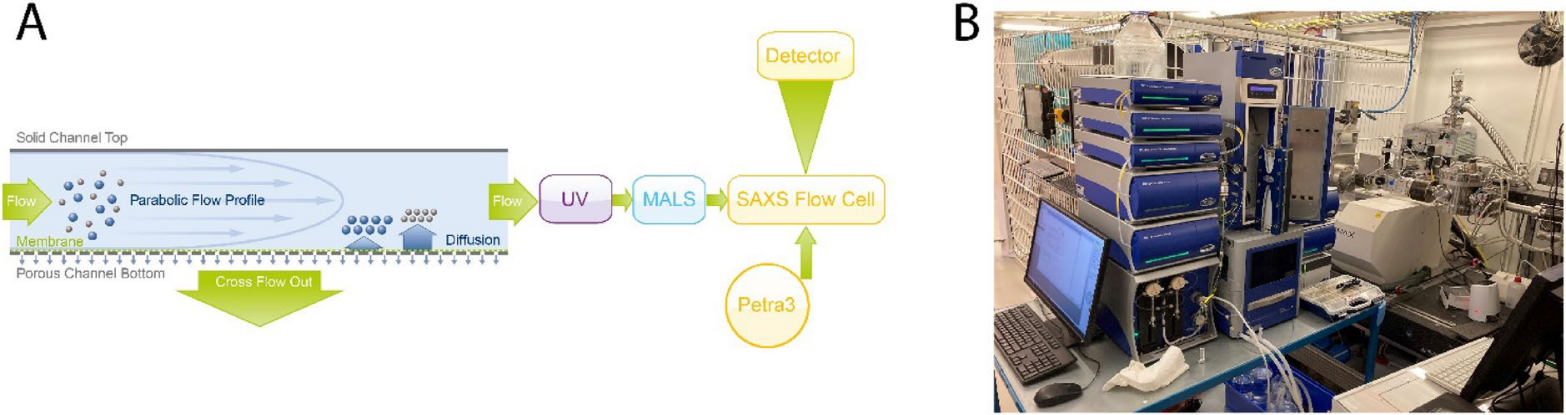
Overview of multi-detector AF4 setup. (**A**) Schematic overview of the set-up and the organization of different modules. After injection, the particles within the sample are fractionated by size in the AF4 channel. UV–Vis and multi-angle light scattering (MALS) detectors are placed before the flow-through SAXS capillary at P12 (PETRAIII) for characterization. (**B**) AF4- SAXS instrumentation as placed in the experimental hutch at the P12 beamline. AF4 setup with fractionation channel (left) connected to the P12 SAXS flow cell in-line (right). Sample separation and data collection are initiated remotely from the control hutch.

Here, for the first time, we describe an approach of directly coupling AF4 to SAXS (AF4-SAXS) to thoroughly characterize nanoparticulate pharmaceutical products. A fully equipped AF4 instrumentation, comprising UV absorption and MALS detectors for analyzing the nanoparticles, was set up directly at the BioSAXS beamline, allowing to obtain the full information from classical, multi-detector AF4 with subsequent in-line SAXS measurements from the same particles (Fig. 1).

We selected bovine serum albumin (BSA) as a model protein to represent the conditions for analyzing protein products for pharmaceutical applications (biologics), where we regarded the separation capacity from oligomers and aggregates, and compared the quality of the data from the monomer with results from the cuvette and SEC-separated measurements.

As a test system for the investigation of nanoparticulate samples by AF4-SAXS, we have chosen lipoplex (LPX) formulations. These LPXs can be tailored to display very high targeting selectivity to specific organs (spleen, lung), depending on the manufacturing protocol (e.g. mixing ratio between mRNA and cationic liposomes)^30–32^. Here we have selected LPXs with high targeting selectivity to lymphatic organs, such as spleen^30,32^, which are assembled with an excess of mRNA. Such LPXs are currently undergoing late-stage clinical trials for cancer immunotherapy^30,31,33^. With their complex composition, consisting of a fraction of free mRNA in coexistence with polydisperse LPX nanoparticles, they are an ideal test system for evaluation of the method.

By applying sophisticated data analysis, we were able to accurately quantify the respective fractions in the formulations, to determine the absolute size distribution profiles of the LPX nanoparticles formulations, and to derive important size-related parameters such as the size-dependent structure and composition of the particles including the quantification of mRNA molecule per LPX. The method can be applied to all the above-mentioned nano-scaled pharmaceuticals as well as samples from life sciences or environmental sciences. In the context of pharmaceutical products, such novel, quantitative insight is of great relevance, for example, for formulation development, identification of critical product parameters, evaluation of manufacturing processes, evaluation of comparability, and product quality control in general.

## Results

### Application for proteins and biologics: BSA as a model protein

Control of aggregation state is of outstanding importance for quality control of biologics. Understanding protein misfolding as well as oligomerization processes are essential for optimal process design and formulation development^34,35^. Elucidation of oligomerization processes and the formation of aggregates has additionally become a hot topic ever since their role in the manifestation of various neurodegenerative diseases has become evident^36^. Furthermore, the preparation of monodisperse samples is essential in order to generate indicative data in life sciences and structural biology^25^.

We demonstrated the overall functionality of the setup for analyzing biologics by testing the separation of the model protein BSA. BSA is an excellent model for studying the aggregation mechanisms of globular proteins^37^ with its known formation of oligomeric states (monomeric, dimer, and higher oligomers including aggregates) under various experimental conditions.

**Figure 2:**
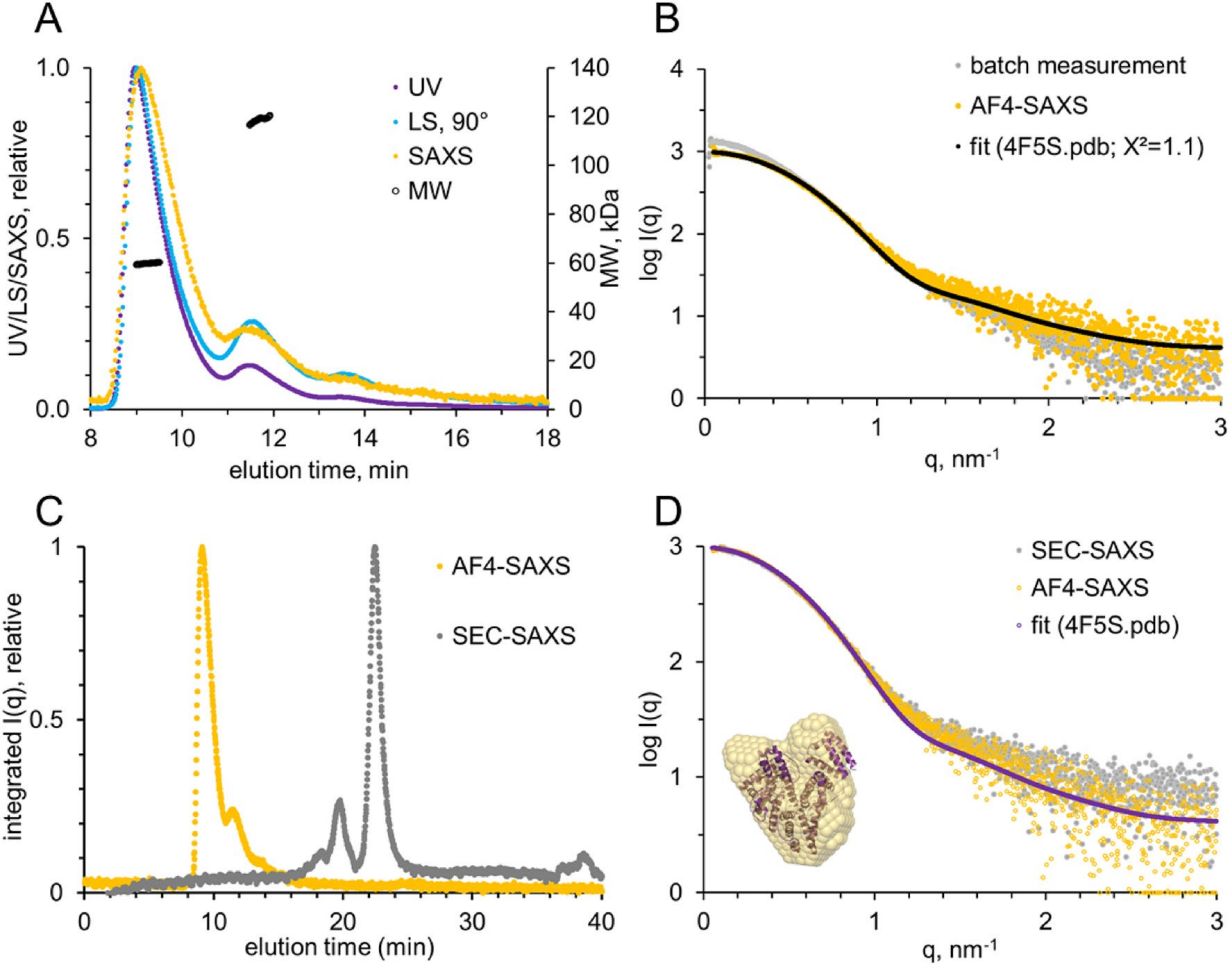
Set-up and system validation with the model protein BSA. SAXS data were collected from either AF4-coupled SAXS or SEC-coupled SAXS after injection of 40 µL BSA in PBS at 5 mg/mL. (**A**) Multi-detector overlay. MW distribution across the peaks suggests successful separation into monodisperse size fractions (monomers, dimers and even higher oligomeric states). (**B**) Comparison of scattering profiles from static measurement (batch, grey) and AF4-SAXS (orange). The fit of the theoretical scattering of the crystal structure is indicated (black). (**C**) Comparison of elution profiles from AF4-SAXS (orange) and SEC-SAXS (grey). Note, with SEC first the larger species elute, in AF4 the opposite is observed. (**D**) The derived SAXS curves from SEC-SAXS (grey) and AF4-SAXS (orange) are in good agreement with each other and can be fitted with the theoretical scattering data of monomeric BSA (purple, 4F5S.pdb, with χ^2^ = 1.1 for the AF4-derived scattering profile). The inlay shows the generated ab initio model as orange spheres overlaid with the cartoon representation of the crystal structure.

BSA was successfully fractionated with AF4, and the elution profiles collected with all detectors (UV_280_, MALS, SAXS) were in good agreement (Fig. 2A). Note, in comparison to UV and LS_90°_ data, the SAXS elution profile shows slight band broadening due to a lower separation resolution because of the larger width of the SAXS flow cell. Nevertheless, the comparison to the data collected without fractionation (Fig. 2B; grey curve) clearly shows that larger species were removed from the sample through the separation process: The decrease in scattering at low q along with the decrease in radius of gyration, R_g_ (from 3.4 ± 0.1 nm to 2.9 ± 0.1 nm), maximum distance, D_max_ (from 13 ± 0.4 nm to 9.1 ± 0.2 nm), and the molecular weight (MW) estimate (based on the volume of correlation from 82 ± 8 kDa to 69 ± 7 kDa) are all in line with the scattering of monomeric BSA as opposed to the mixture containing roughly 10–20% dimeric and larger oligomeric fractions (supplementary Table 1). The constant MW distributions across the main elution peak as well as the second peak further confirm the successful separation into monodisperse fractions (Fig. 2A).

The obtained SAXS data frames corresponding to monomeric BSA displayed a good fit to the theoretical scattering curve derived from the atomistic crystal structure (χ^2^ = 1.1; 4F5S.pdb, Fig. 2D). In addition, the ab initio model derived from the purified scattering curve overlays well with the crystal structure.

Comparing AF4-SAXS and SEC-SAXS scattergrams (Fig. 2C) the opposite separation characteristic becomes obvious: While with AF4 smaller particles elute first, the contrary is the case for SEC. Both methods are able to separate the monomer from the oligomers properly. Data collection time, sample consumption and data quality are comparable for AF4-SAXS and standardised SEC-SAXS data mode which is commonly used to study volume mixtures of biological macromolecules at dedicated SAXS beamlines at synchrotron radiation (SR) facilities (Fig. 2C)^21^. The elution of the smaller component before the larger particles including aggregates that are often prone to radiation damage and subsequent capillary fouling, highlights the single benefit of AF4 in this case of protein oligomer separation.

### Complex nanoparticulate pharmaceutical products: mRNA LPX formulations

**Figure 3:**
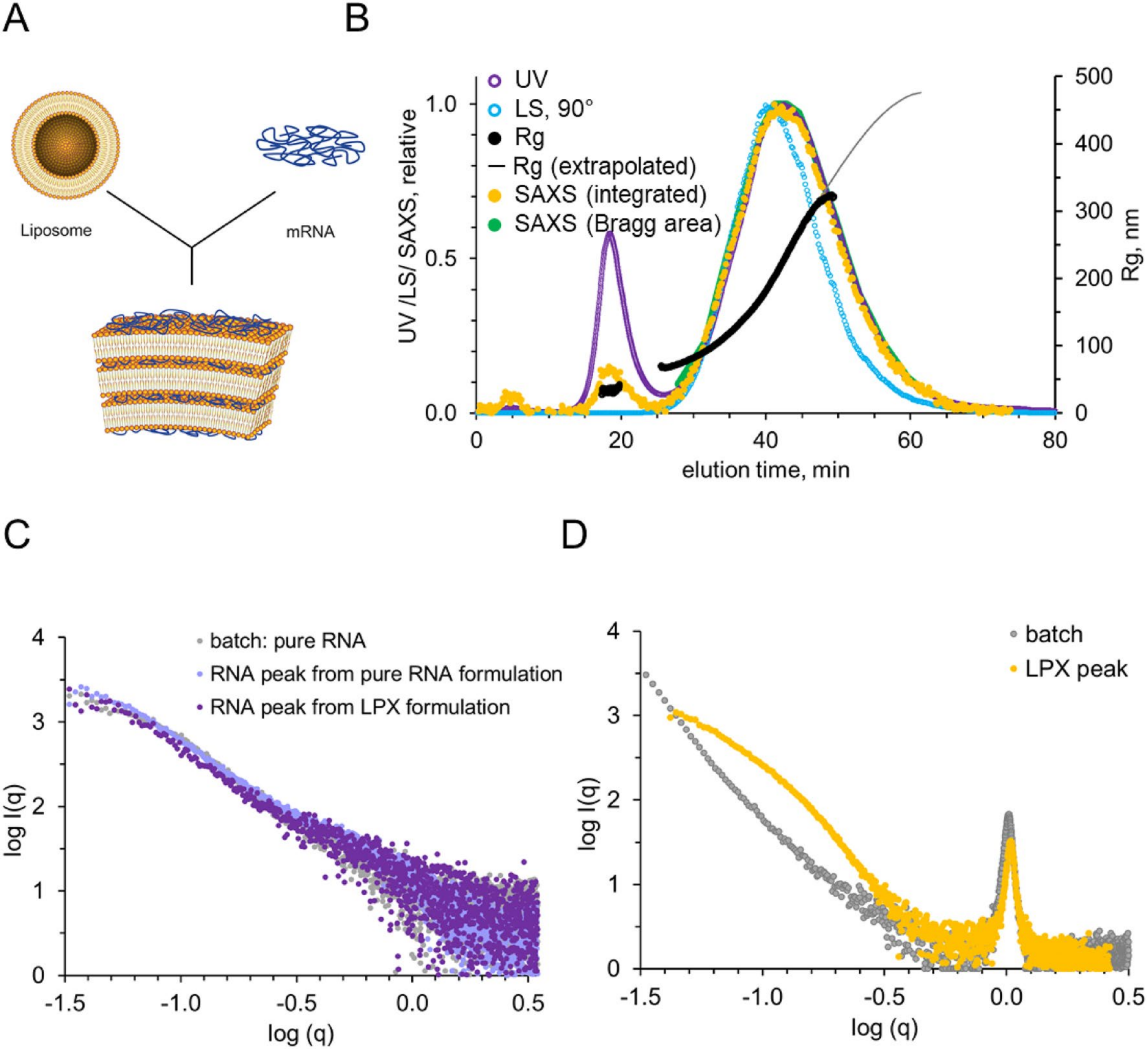
AF4 coupled SAXS analysis of LPX fractionated sample. (**A**) Self-assembly of mRNA LPXs by mixing of anionic mRNA (blue) with premanufactured cationic liposomes. (**B**) AF4-SAXS analysis of LPX formulation versus elution time with an overlay of fractograms derived from UV, LS, and SAXS including derived R_g_ values. The first peak, around 18 min, results from free mRNA, and the second peak, around 40 min, is from the mRNA lipoplex nanoparticles. (**C**) SAXS patterns derived from pure mRNA (grey) in a batch measurement compared with measurements of RNA after fractionation with AF4 (purple). (**D**) Comparison of a batch LPX sample with a purified LPX sample after fractionation. Note the much lower slope of the fractionated sample towards q = 0, indicative for successful separation of other particulates.

As a test system for the investigation of nanoparticulate samples by AF4-SAXS, we have chosen LPX formulations, obtained by self-assembly between cationic liposomes and mRNA (Fig. 3A). For the present measurements, we specifically selected LPX systems which were formed with an excess of mRNA, resulting in negatively charged, polydisperse, LPX nanoparticles in coexistence with a fraction of free, unbound mRNA^30–32^. cryo-TEM measurements have demonstrated lamellar internal organization inside the LPXs^30^. This test system comprising polydisperse nanoparticles as well as negatively charged, free mRNA, is ideal to challenge and evaluate the AF4-SAXS approach while delivering information on the colloidal nature of such formulations, which is essential for assessing biological activity and quality. Note, that due to the opposite charge of the pure DOTMA:DOPE vesicles, conducting a direct comparison of liposome size fractions without mRNA under identical experimental AF4 conditions was not feasible in this session.

The LPXs were injected, and the size-separated fractions were measured in-line by the subsequent detection systems. AF4 allowed for the effective separation and quantification of the two distinct fractions within the LPX formulations, as clearly demonstrated by the multiple elution traces obtained from the respective detectors (Fig. 3B). UV absorption measured at 260 nm, commonly used for mRNA quantification, revealed an initial peak, attributed to the presence of free mRNA, and a second, larger, peak corresponding to the LPX nanoparticles (Fig. 3B, purple trace). For the LPX peak it has to be kept in mind, that, further to UV absorbance, size-dependent scattering of the particles contributed to the signal. The X-ray scattering intensity (yellow trace), which is a measure of the absolute amount of material (Supplement Fig. 1), resulted in peaks for mRNA and LPXs at the same elution times as the UV peaks, however, the mRNA peak was relatively lower than in UV, in accordance with the above conjecture, that the UV signal of the LPXs was indeed influenced by scattering in addition to UV absorption (note, that the signals were scaled to 1 for the LPX peak). Light scattering at 90° (light blue trace) revealed also a pronounced peak for the LPXs, while the signal for the free mRNA was even lower relative to the LPX peak (as expected from the size dependence of scattering intensity).

Although the LS signal was weak, the radius of gyration (R_g_) and the molecular weight of the mRNA could be calculated (Fig. 3B). A relatively constant R_g_ of about 25 nm, in accordance with a macromolecule with a discrete molecular weight (black horizontal line under the mRNA peak, Fig. 3B), was obtained. MALS allowed us to experimentally determine MW (~ 475 ± 50 kDa) which is well in accordance with the theoretical value of ~ 400 kDa. This proves that the first peak resulted in fact from pure mRNA, without further molecular moieties (as, for example, lipid material) bound to it.

For the second peak, resulting from the LPXs, a clear separation of the particle size as a function of elution time from about 80 nm to about 470 nm (R_g_) was determined (black line under the LPX peaks). This indicates the validity of the AF4 method for analysis of the LPX nanoparticles in the present samples (see also for complementary DLS measurements in Supplement Fig. 2).

SAXS offered direct structural insight into the respective fractions (Fig. 3C + D). Curves for the mRNA were indicative of unstructured random coil conformation (Supplement Fig. 3): the pair distance distribution function, p(r), displayed a skewed, extended shape, and in the Kratky plot a plateau towards large q, without intermediate maximum was obtained. With this structural organization, the mRNA differs substantially from the BSA which is characterized by a more globular packing. This highlights the strength of solution SAXS in offering structural information and its capability to assess compact, extended as well as flexible systems. The MW estimations from the SAXS curves were in accordance with the mRNA monomer found by MALS (440–450 kDa).

The LPX scattering curves were dominated by a Bragg peak (1 nm^-1^), indicative of a lamellar organization of mRNA inserted into repeating lipid bilayers inside the nanoparticles^38^. The intensity at the q range below the peak displayed monotonous decay, where the slope of − 3.63 was in accordance with the presence of compact particles with smooth surface (Porod analysis, see Methods Part for details).

Notably, the fractionated LPX curves, as opposed to the bulk measurement, did not exhibit an increase in intensity towards q = 0, in agreement with the successful fractionation to rather monodisperse size fractions and removal of mRNA and any larger moiety. With that successful separation of the polydisperse LPXs into monodisperse and uniform moieties, further, quantitative data analysis was enabled.

### Quantitative size distribution and size-dependent parameters

One aim of the present study was to obtain quantitative, absolute size distribution profiles for the particulates. This was made possible by in-line coupling of SAXS to classical multi-detector AF4, and a joint analysis of the data from the different detectors. For quantification of the free mRNA, UV absorption at 260 nm as used as well for cuvette measurements could be applied. The mRNA absorption dominates the UV signal of the material under the LPX peak, but for quantitative analysis as well scattering from the particulates has to be taken into account, which depends on particle properties such as size, shape and refractive index gradient. With such correction the concentration of RNA as a function of elution time is given, and with the knowledge of the lipid-to-mRNA ratio in combination with the size information from MALS, the absolute mass fraction of the particles at that size can be calculated.

X-ray scattering depends only on the electron density (gradient) and is therefore advantageous for the determination of absolute concentrations. In the standard approach for the determination of molecular weight from SAXS, the scattering intensity is extrapolated to zero scattering angle (I(0)), and with known concentration and electron density gradient, MW is given. Here we have used the particle size at a given elution time obtained from the MALS measurements and combined it with the SAXS scattering intensity to obtain the concentration.

With the applied settings for the experiments here, absolute determination of I(0) was hampered due to the missing data at very low q (Supplement Fig. 4). However, as the total concentration of the particulates is known (see Methods section) one can as well use the relative signal (e.g., from lowest measured q) and normalize over the known total concentration of LPX matter. This is also justified by the observation, that the fundamental characteristics of the particles did not change over the peak, and therefore the fundamental shape of the scattering curves should not substantially change.

**Figure 4:**
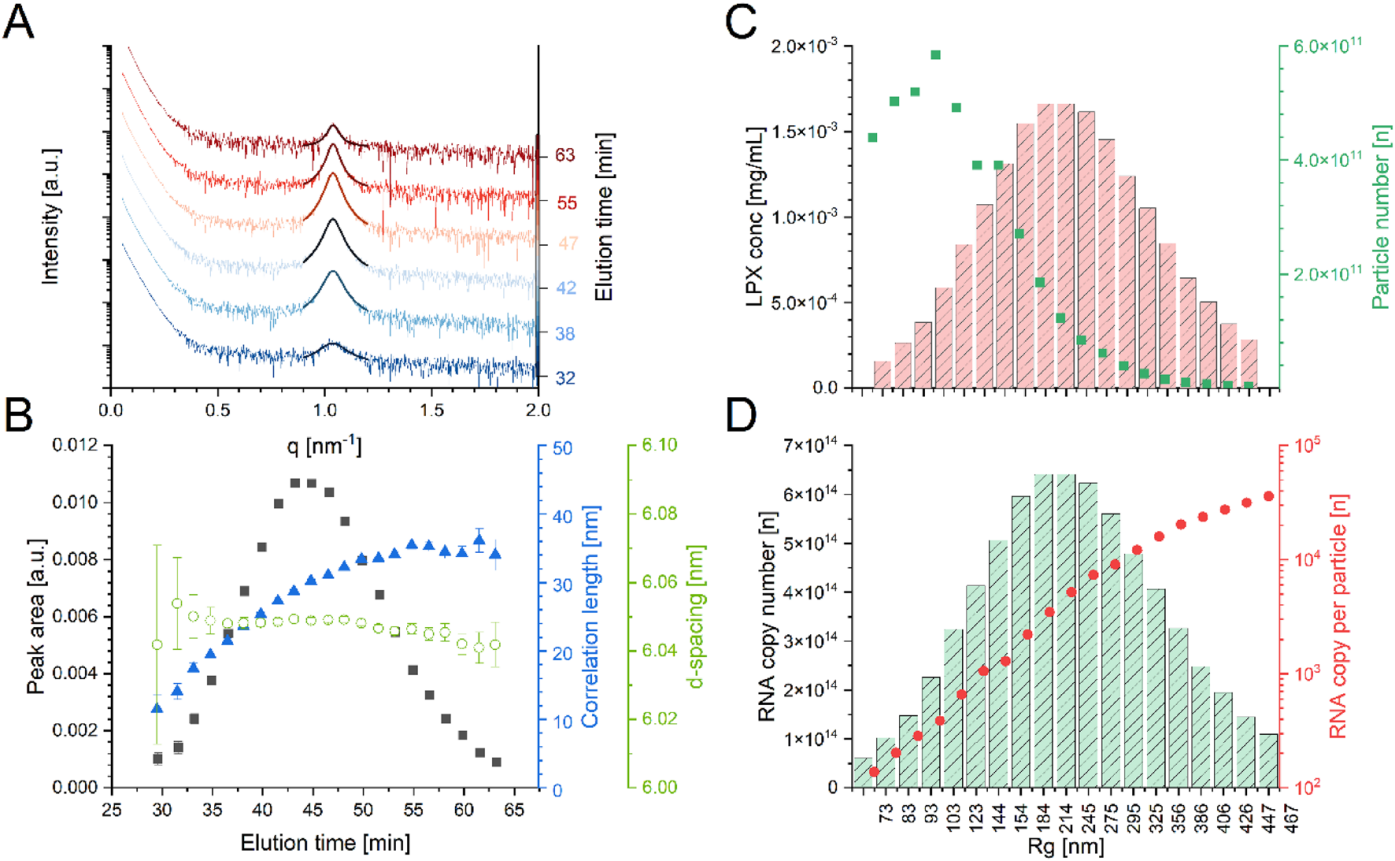
Detailed analysis of the fractionated samples. Adding AF4 separation directly before the SAXS data collection allows the correlation of SAXS derived information as a function of size (elution time). (**A**) SAXS patterns derived from different size fractions with respective Lorentzian fits (shifted on y-axis by increasing elution time). (**B**) Derived parameters from peak analysis of the individual scattering curves (d-spacing in green; correlation length in blue, peak area in black). (**C**) Absolute material concentration (LPX conc. in mg/mL) derived from SAXS signal for LPX peak (red bars) and the calculated absolute number of particles as a function of radius of gyration (R_g_) derived from MALS (green dots). (**D**) The total number of mRNA copies calculated from SAXS traces in certain fractions of different particle sizes (R_g_ from MALS) (green bars) and calculated mRNA copies per particle of size segment (red dots).

As a second piece of information from the SAXS curves, one can analyze the evolution of the Bragg peak, which is measured for the LPX particles. Qualitative inspection of Bragg peaks from different LPX elution fractions (Fig. 4A) indicates, that only the area of the consecutive peaks changed over the elution time, while the peak position and shape were relatively similar. Therefore, the LPX nanoparticles consisted of the same type of ordered material with a characteristic d-spacing of about 6.0 nm independent of their size (which ranged from 80 to 470 nm), with the same lipid-to-mRNA stoichiometry for all particles (internal data, to be published).

We used Lorentz functions to quantitatively determine peak position, peak width, and peak area to calculate d-spacing, correlation length, and the total amount of ordered material (formalism in Methods section; Fig. 4B, Supplement Table 2). While the d-spacing (green), in fact, was basically similar over the elution time of the whole LPX peak, the correlation length (blue) increased slightly, in accordance with increasing size of the LPX stacks inside the larger particles. The peak area, indicative for the amount of ordered material, showed a similar peak shape over time as the UV and total SAXS signal (Fig. 4B, black trace). Apparently, all lipoplexes were characterized by the same type of internal order, with a continuous increase of organization with particle size. No fundamental change of structural characteristics at a certain size occurred, as also obvious from the plot of the peak area divided by peak with (Supplementary Fig. 5)^39^.

Supplement Figure 4 shows the correlation of Bragg peak area, I(q), and UV signals as a function of elution time. One can see that the trace for detected forward scattering behaves like the UV absorption (and scattering), indicating that at the current setting the ‘large particles’ become ‘invisible’ in the Guinier analysis, and confirming the above conjecture of constant stoichiometry for all particles. We consider the ordered material as the one which is biologically active, therefore, we used the Bragg peak area for quantitative analysis of relative mass fractions. We obtain the amount and copy number of active (lipoplexed) mRNA in the particles, and, with the constant lipid stoichiometry, the total amount (mass) of the lipoplexed material at any given size fraction.

Valuable information can be drawn from this analysis: product characteristics such as LPX concentration in µg/mL, number of particles (Fig. 4C, red columns, green dots), mRNA copy number, and the mRNA copy number per particle (Fig. 4D, green columns, red dots) are obtained as a function of the particle size (formalism see Methods section). The largest amount of LPX material (in µg/mL) is present in the form of particles of about 200 to 250 nm, while the highest number of particles is at the size fraction of about 100 nm (see methods and materials for calculations). However, not only the number of mRNA copies per particle, but also the total number of copies is much lower at the 100 nm than for the 200 nm fraction (Fig. 4D).

Furthermore, several other parameters which are relevant for the quality of particulate products (in the size range of the wavelength of light and above using counting methods), such as median and mean values (D10/D50/D90) with respect to mass concentration, particle concentration, and other parameters, can be readily derived.

## Discussion

Quantitative determination of size-dependent parameters for nano-scaled colloidal systems is still an unmet need in many fields, including pharmaceutical research and development, as well as life and environmental sciences. This is, in particular, the case for small particles (smaller than the wavelength of light, when the particles are too small to be determined by counting methods such as laser light obscuration). Although some methods to generate size distribution profiles are available, they generally provide only qualitative information, while it is still difficult to obtain absolute data.

Here we addressed this challenge by in-line coupling of classical multi-detector AF4 with synchrotron SAXS measurements, methods which usually are only applied separately from each other. SAXS signals per se provide information on absolute concentrations. With the advantage of the high flux synchrotron beam allowing focusing on the small flow-through capillary, measuring low concentrations of solutes, and adjusting q from the USAXS range to WAXS, information on particle size, shape, packing (conformation) and internal order and crystallinity is obtained. Such information can be generated at synchrotron sources regularly within short exposure times and for a variety of different sample types.

For analysis of SAXS data, it is important to provide samples as much as possible in a single state (size, shape conformation), which is realized by the AF4 separation in the best possible manner. By in-line coupling of AF4 with simultaneous detection applications of standard methods (UV, MALS, and DLS) and SAXS comprehensive data on the fractionated samples are quantitatively obtained.

The high adaption of the use of SEC-SAXS by the structural biology community^20,21^ clearly demonstrates the advantage of SAXS analysis with in-line separation techniques. With using AF4 for size separation, we can eliminate certain drawbacks of SEC. Pressure issues and unfavorable shear forces often cause the biggest problems while the samples pass through the column matrix (stationary phase). In contrast, in the AF4 separation technique, a liquid-filled channel is used and thus these effects are not observed. Especially for weak-binding complexes these shear forces may affect the complexation state and, thus, cannot be used to get information on the native conformation. Other drawbacks of SEC include limitations in the choice of buffer and additives for a successful SEC separation. AF4 allows adjustment of buffer and flow parameters in a wider range, and therefore, tailored protocols for different types of particulates can be developed.

This makes AF4-SAXS particularly interesting for the investigation of complex systems, allowing to reveal quantitative profiles of broadly disperse systems, and to determine different fractions of nanoparticulates concerning degree of loading, structure, or functionalization. The capability of AF4 to separate molecular drugs or small proteins from nanocarriers, and nanocarriers from particles of micrometric size, is a major advantage for investigation of drug-loaded nanoparticles^28^. Also, different protein moieties with similar properties can be separated and analysed, with applications to pharmaceutically relevant proteins, as well as to basic structural biology research.

By optimizing the SAXS capillary in conjunction with the AF4 system, the scope of application can be also extended to low concentration samples. Thus, our upcoming experiments will entail dedicated beamtime sessions to fine-tune capillary lengths and diameters (all while maintaining the necessary backpressure for consistent AF4 fractionation). Furthermore, implementing the co-flow system could present an elegant prospect for mitigating the undesired dilution^40^.

Here we have demonstrated the capabilities of AF4-SAXS for analyzing pharmaceutical formulations comprising lipoplex nanoparticles in coexistence with free mRNA. With our set-up of in-line coupling multi-detector AF4 SAXS measurements, we quantified and characterized the two fractions and obtained in-depth information on LPX particle characteristics such as drug-load and structure as a function of size.

While other approaches investigate this with confocal spectrometry methods by using labeled mRNA, lipids, and fluorescent dyes^41^, our setup allows us to investigate formulations as they are used in clinical studies without further modifications. Additionally, it applies to other nanoparticulate formulations with different payloads as mRNA.

This allows us to apply the method for investigation of nanoparticles from various scientific fields, which either encounter issues regarding sample monodispersity and homogeneity or, by their nature, are broadly polydisperse and consist of different moieties. In pharmaceutical sciences, the method provides an invaluable contribution to characterizing the various types of colloidal nano-scaled products including liposomes, lipid nanoparticles, polymer nanoparticles as well as inorganic systems like SiO_2_ or metal particles. As well, information on large molecules such as DNA, RNA therapeutic proteins (biologics), or dissolved polymers can be obtained. As shown in the present measurements for samples comprising a disordered biopolymer together with polydisperse nanoparticles, information on complex systems, can be obtained. The fractions in samples comprising different nanoparticle or colloidal moieties, or having different structures, shapes or molecular organization, can be quantitatively assessed. Further to pharmaceutical products, samples from life sciences, other industrial nanoparticles, and bio-eco particulate systems can be measured. For example, the separation of particles from environmental studies through AF4 coupled to other techniques such as Raman spectroscopy has allowed comprehensive studies of nanoplastics^42^. Obviously, here too, the additional information gained by X-ray scattering can deliver accurate information for example on the size, shape, and surface of environmentally relevant nanoparticles.

## Conclusion

In summary, we have introduced a novel and versatile approach for obtaining quantitative, size-dependent information on nano-pharmaceuticals and colloids. As demonstrated with a model protein and complex mRNA lipoplex formulations, the method enabled the successful separation of the respective fractions comprised inside the products and, thus, allowed for their unequivocal quantification and characterization. The data are highly relevant in pharmaceutical development from a quality and safety perspective. The method has the potential to become a basic tool for the characterization of all these systems.

## Methods

### LPX formulation

LPX were formed by mixing cationic liposomes containing (R)-N,N,N-trimethyl-2-3-dioleyloxy-1-propanaminium chloride (DOTMA) and 1,2-dioleoyl-sn-glycero-3-phosphoethanolamine (DOPE) (both Merck KGaA, Darmstadt, Germany) lipids with the negatively charged messenger RNA (mRNA), using the semi-automated microfluidic path process, internal protocols based on previously described batch protocols^31,32^. Briefly, for incubation with the mRNA, a pumping system was used to mix equal volumes of two aqueous phases, comprising mRNA and liposomes. The mRNA was synthesized at BioNTech using internal protocols. The liposomes consisted of DOTMA and DOPE in a molar ratio of 2:1. Concentrations were adjusted to achieve a molar (charge) ratio between the positively charged DOTMA molecules in the liposomes and the negatively charged nucleotides of the mRNA of 0.65 to 1.

### AF4 separation

Ultrapure water (UPW) was obtained from a Milli-Q system (Integral 5 system, Merck KGaA, Darmstadt, Germany). A carrier liquid of 10 mM HEPES (C. Roth GmbH Co. KG, Karlsruhe, Germany), 5 mM sodium chloride (Avantor Performance Materials Poland S.A.) and 0.1 mM tetra sodium salt of ethylenediaminetetraacetic acid (EDTA) (Merck KGaA, Darmstadt, Germany) was prepared and the pH was adjusted to 7.4 using 2 M NaOH (Chemsolute, Th. Geyer GmbH Co. KG, Karlsruhe, Germany). For system qualification and proof-of-concept study, a PBS buffer solution with 10 mM phosphate salts (potassium dihydrogen phosphate and sodium hydrogen phosphate salts, both obtained from Th. Geyer GmbH Co. KG, Karlsruhe, Germany), 137 mM sodium chloride, 2.7 mM potassium chloride (C. Roth GmbH Co. KG, Karlsruhe, Germany) at a pH of 7.4 was produced. Afterwards, all carrier liquids were filtered with a vacuum filtration unit through a 0.1 µm pore membrane (Durapore, Merck Millipore Ltd., Tullagreen, Ireland).

For the normalization of the MALS detector angles a polystyrene nanoparticle (PS-NP) size standard with a nominal diameter of 62 nm ± 4 nm at a concentration of 1% (w/w) (Nanosphere™ Size Standard 3060A, Thermo Fisher Scientific, Waltham, MA, USA) was fractionated. A carrier liquid of 0.2% (v/v) NovaChem (Postnova Analytics (PN), Landsberg am Lech, Germany) was used for the fractionation of PS-NP.

No sample preparation or dilution was performed prior to fractionation. The samples were fractionated by an AF4 system (PN AF2000 MT). Additionally, the system included an autosampler (PN 5300) and slot outlet function (PN 1650). A semi-preparative frit-inlet AF4 (Fl-AF4) channel (shoulder width 50 mm, tip width 5 mm, tip-to-tip length 277 mm) was equipped with a polyethersulfone PES membrane with 10 kDa molecular weight cut-off and a Mylar spacer of 350 µm height. Preliminary offline tests using this channel suggesed that this configuration can accommodate the injection of up to 150 µg of mRNA. The temperature of the autosampler was set to a constant temperature of 4 °C. The system was hyphenated to a UV detector (PN 3211) and a MALS detector (PN 3621, 21 angles). The UV absorbance wavelength was set to 260 nm. The MALS detector angles were normalized with respect to 90° using a fractionated 62 nm PS-NP size standard. The MALS was directly connected to the SAXS flow capillary using a minimal tubing length with an ID of 250 µm. MALS data of LPX samples were evaluated by applying a 1st order Berry model fit to at least 9 active angles^15^. The number of forward scattering angles was limited due to the strong scattering contribution of the larger LPX particles. As a result, the derived R_g_ distribution ranged up to around 320 nm with a squared correlation coefficient R^2^ of the fit higher than 0.99.

The injected volume varied from 250 µL up to 1000 µL. The fractionation method consisted of an exponential cross flow decay covering the sample size range in one single fractionation run. The slot outlet function with a flow rate of 0.2 mL min^−1^ was applied to concentrate the sample constituents after fractionation. Moreover, a sufficient rinse step of 12 min was implemented to minimize carryover and potential memory effects.

To test the suitability of the semi-preparative FI-AF4 channel all samples were also characterized on a standard FI-AF4 channel with the same tip-to-tip length, but smaller width of 20 mm. Here, all samples were analyzed in triplicate injecting only 40 µL to avoid potential overloading effects. The extrapolation of the R_g_ distribution (Fig. 1C) was carried out based on the last-mentioned measurements using a polynomial fit with excellent agreement. Here the aforementioned Berry 1st order model fit was applied to the scattering intensities derived from at least 11 active scattering angles. Instrument control and data evaluation were performed by the NovaFFF AF2000 software (PN, Version 2.2.0.1) and Excel (Microsoft Corporation, Office 2013). To comprehensively characterize the samples the MALS detector was connected to a Zetasizer Nano S (MALVERN Panalytical Instruments Ltd., Malvern, UK) in flow mode to derive hydrodynamic sizes from DLS.

### SAXS data collection

AF4-SAXS data was collected at the P12 bioSAXS beamline of the European Molecular Biology Laboratory (EMBL) at the PETRA III synchrotron, DESY Hamburg (Germany)^43^, using an incident beam size of 200 × 110 μm^2^ (full width at half maximum). The eluent of the employed fractionation technique was passed through a 1 mm quartz capillary held under vacuum.

The SAXS data were recorded on a Pilatus 6 M area detector (Dectris) at a sample-to-detector distance of 3 m and the wavelength *λ* = 0.155 nm (X‐ray energy 8 keV). Series of individual 1 s exposure X‐ray data frames were measured from the continuously flowing column eluate across one column volume. The 2D SAXS intensities were reduced to *I*(*q*) versus *q* using the integrated analysis pipeline SASFLOW. The *q*‐axis was calibrated with silver behenate, and the resulting profiles were normalized for exposure time and sample transmission. This results in one‐dimensional scattering intensity curves *I*(q) presented as functions of momentum transfer

1 $$q = \frac{{4\pi \cdot {\text{sin}}\left( \theta \right){ }}}{\lambda }$$

where 2*θ* is the scattering angle and *λ* is the X‐ray wavelength.

Additional data was collected with an automated sampler changer and in SEC-SAXS mode. Data collection details are summarized in Extended Data Table 1.

The 1D scattering curves were further analysed with Chromixs–allowing us to define the frames of interest as well as frames for buffer subtraction.

DATTOOLS were used to average data and facilitate downstream processing as well as increase the overall signal-to-noise ratio.

### SAXS analysis

Data transformation and analysis were performed using QtiPlot 1.0.1 (IONDEV, Romania) and the ATSAS package (EMBL Hamburg, Germany)^19^. The LPX SAXS curves were characterized by a single Bragg peak, indicative of the repeat order inside the particles. The Lorentzian fit functionality in QtiPlot 1.0.1 was utilized for peak fitting of the Bragg peaks in the SAXS curves

2 $$I\left( q \right) = I_{0} + \frac{2A}{\pi } \cdot \frac{w}{{4 \cdot \left( {q - q_{c} } \right)^{2} + w^{2} }}$$

with *I(q)* being the scattering intensity, *I*_*0*_ the baseline intensity at q = 0, extrapolated from the peak position, *A* the peak area, *w* the peak width (FWHM), and *q*_*c*_ the peak position. From the peak position *q*_*c*_ the corresponding repeat distance of the scattering moiety *d* was calculated using the Bragg equation^44^

3 $$d = \frac{2\pi }{{q_{c} }}$$

From the peak width, w, the correlation length, ξ inside the ordered stacks was calculated. Assuming liquid crystalline organization, ξ can be defined as the distance, at which the positional correlation decays to the value 1/e and is given as^45^:

4 $$\xi = \frac{2}{w}$$

The area of the peaks, A, was taken as a measure for the total amount of material present in the respective state of the organization.

Additional information can be revealed by analyzing the intensity decay using the power law as shown in the following equation:

5 $$I\left( q \right) = { }I_{0} \cdot q^{ - x}$$

The so-called Porod exponent *x*, is a measure for the fractal dimension of the particle surface. For an ideal flat interface, the Porod exponent is 4 (Porod law) while with increasing roughness (more specifically increasing fractal dimension) the Porod exponent decreases^46,47^.

For the processed BSA scattering profiles further analysis was performed including ab initio reconstruction (DAMMIF^48^) and model fitting (CRYSOL^49^). Basic structural parameters and MW estimates were determined with the tools implemented in PRIMUS.

The BSA data were deposited in the Small Angle Scattering Biological Data Bank (SASBDB). The accession codes are listed in Extended Data Table 1.

### Formalisms for the quantitative determination of size-dependent parameters

#### . Size-dependent mass fractions of LPX nanoparticles

For the calculation of the absolute mass of LPX as a function of size, we used the following premises:

- *Total concentration of mRNA* in the lipoplex formulations (in our measurements 0.15 mg/mL)
- *Mixing ratio (charge ratio) between liposomes and RNA for lipoplex* formation (here: DOTMA/RNA = 0.65, using 330 Da as the average mass of the negatively charged nucleotide and assuming one positive charge per DOTMA molecule)
- *Liposome composition* (here DOTMA/DOPE in a molar ratio of 2 to 1, with the MW of DOTMA-cation of 635 Da and of (zwitterionic) DOPE of 740 Da. The total lipid MW per charge was therefore 1005 Da (635 + 740/2)
- *Composition of lipoplex nanoparticles* (here we use a one-to-one stoichiometry between DOTMA and RNA nucleotide, as previously determined by independent measurements, to calculate the composition of the lipoplexes^50^)

Therefore, in the LPXs, each mRNA nucleotide of 330 Da was bound to lipids with 1005 Da, nucleotide and lipids having then a total MW_nucleotide+lipids_ of 1335 Da.

The total LPX concentration, *c*_*LPXtotal*_ in mg/mL, was calculated using the following general equation:

6 $${\text{c}}_{LPXtotal} = \left( {c_{RNAtotal} - c_{RNAfree} } \right) \cdot \frac{{MW_{nucleotide + lipids} }}{{MW_{nucleotide} }}$$

where *c*_*LPXtotal*_ is the total concentration of LPXs (mRNA + lipid), *c*_*RNAtotal*_ the total mRNA concentration in the formulation, *c*_*RNAfree*_ the free mRNA concentration, *MW*_*nucleotide*+*lipids*_ the molecular weight of the lipids and nucleotide complexes, *MW*_*nucleotide*_ the molecular weight of nucleotide for LPXs.

The experimentally determined value for the free mRNA concentration was 0.045 mg/mL, corresponding to about 30% of the total amount, which is in very good accordance with the expectation (35%). For *c*_*LPXtotal*_, one obtains:

7 $$c_{LPXtotal} = \left( {0.15 - 0.045} \right) \cdot \frac{1335}{{330}} = 0.425\left[ {\frac{{{\text{mg}}}}{{{\text{mL}}}}} \right]$$

From the experimental data, the peak area of the LPX peak from AF4 measurements was integrated (more specifically, the Bragg peak area from Lorentz fitting was integrated). This value was normalized with respect to the calculated total LPX content, *c*_*LPXtotal*_, given above, and the normalization factor was used to calculate the differential absolute LPX concentration, c_LPXdif_, as a function of size.

#### Size-dependent particle number and composition

Particle numbers as a function of size were calculated from the above obtained absolute mass fractions by using simplified models for particle shape. As LPX particles are known to be globular and compact entities, they were approximated as solid spheres with the volume:

8 $$V = \frac{4}{3}\pi R^{3}$$

The experimentally determined radius of gyration, R_g_, correlated therefore with the radius, R, of the solid sphere to

9 $$R = \sqrt{\frac{5}{3}} R_{g}$$

With the further approximation of the density, ρ_LPX_, of the material which forms the LPX nanoparticles (1.11 g/mL, using ρ_RNA_ $\approx$ 1.68 g/mL and ρ_lipid_ $\approx$ 1.0 g/mL), the mass per particle, m_p_ is given as:

10 $$m_{p} = {\text{V}} \cdot {\uprho }$$

The number concentration of particles within a given size fraction, n_LPXdif_, is given from the differential LPX concentration c_LPXdif_:

11 $$n_{LPXdif} = \frac{{c_{LPXdif} }}{{{\text{m}}_{p} }}$$

The number of mRNA copies, n_mRNA/particle_, in a single particle is given by the particle mass, m_p_, the molar mass of the lipid-complexed nucleotide, MW_nucleotide+lipid_, the number of nucleotides of the mRNA, n_nucelotide_, and Avogadro’s number, N_A_:

12 $${\text{n}}_{mRNA/particle} = \frac{{m_{p} \cdot N_{A} }}{{MW_{nucleotide + lipid} .n_{nucleotide} }}$$

The total number concentration of mRNA copies in particles at a certain size fraction is given by the concentration fraction, c_LPXdif_, the molar mass of the lipid-complexed nucleotide, MW_nucleotide+lipid_, the number of nucleotides per RNA copy, n_nucelotide_, and Avogadro’s number, N_A_:

13 $$n_{RNA/dif} = \frac{{c_{LPXdif } \cdot N_{A} }}{{MW_{nucleotide + lipid} .n_{nucleotide} }}$$

### Supplementary Information

Supplementary Information.

## Data Availability

The datasets on BSA generated and/or analyzed during the current study are available in the Small Angle Scattering Biological Data Bank (SASBDB), SASDRQ8. The lipoplex SAXS data are accessible under: SASDSH7. No human or other cell lines were used for the present experiments.
