# Supplementary material for: Quantitative size-resolved characterization of mRNA nanoparticles by in-line coupling of asymmetrical-flow field-flow fractionation with small angle X-ray scattering
Source: Sci Rep. 2023 Sep 22;13:15764. doi: 10.1038/s41598-023-42274-z (PMC10516866; doi:10.1038/s41598-023-42274-z)
Supplement: Supplementary file 1 — Supplementary Information. [file 41598_2023_42274_MOESM1_ESM.docx]

**SUPPLEMENTARY INFORMATION**

**Quantitative size-resolved characterization of mRNA nanoparticles by in-line coupling of asymmetrical-flow field-flow fractionation with small angle X-ray scattering**

Melissa A Graewert^1*✉^, Christoph Wilhelmy^2*^, Tijana Bacic^3*^, Jens Schumacher^3^, Clement Blanchet^1^, Florian Meier^4^, Roland Drexel^4^, Roland Welz^4^, Bastian Kolb^2^, Kim Bartels^2^, Thomas Nawroth^2^, Thorsten Klein^4^, Dmitri Svergun^1,5^, Peter Langguth^2^ & Heinrich Haas^2,3,✉^

^1^ European Molecular Biology Laboratory, Hamburg Unit, Hamburg, Germany

^2^ Department of Biopharmaceutics and Pharmaceutical Technology, Johannes Gutenberg-University, Mainz, Germany

^3^ BioNTech SE, Mainz, Germany

^4^ Postnova Analytics GmbH, Landsberg am Lech, Germany

^5^ BIOSAXS GmbH, Hamburg, Germany

*These authors contributed equally

✉melissa.graewert@embl-hamburg.de

✉haashein@uni-mainz.de

**Acknowledgments**

This research was funded by the „Bundesministerium für Bildung und Forschung BMBF“ grant 05K22UM3 and by the “Deutsche Forschungsgemeinschaft DFG” as part of the collaborative research center (CRC) 1066.

**Declaration of interest**

MAG is consultant to BioSAXS GmbH

PL is consultant to BioNTech SE

**Supplement Table 1.** SAXS sample details, data collection, analysis, and 3D modelling details for BSA in solution.

|  | **Batch** | | **AF4 monomer peak** | | |
| --- | --- | --- | --- | --- | --- |
| Sample temperature (°C) | 20 | | | | |
| In beam sample cell | 1‐mm quartz capillary | | | | |
| Sample concentration, mg/mL | 5.0 | | 5.0 (40 ul injection volume). | | |
| In-line separation | n.a. | | semi-preparative frit-inlet AF4 channel  PES membrane (10 kDa MW cut-off)  and a Mylar spacer of 350µm height | | |
| Detector flowrate, mL/min | n.a. | | 0.5 mL/min | | |
| Data acquisition/reduction software | SASFLOW | | |  |  |
| Instrument | P12, Petra3@DESY, 6M-Pilatus detector | | | | |
| Measured *q*-range (nm^-1^) | 0.02 – 5.83 | | | |  |
| Exposure time(s), number of exposures. | 0.050, 20 frames | | 1, 2500 frames (23 averaged) | | |
| Methods/Software | PRIMUS | | CHROMIXS, PRIMUS | | |
| *Guinier Analysis* |  | |  | | |
| *I*(0) ± σ (cm^-1;^ a.u) | 2639.21 ± 3.84 | | na | | |
| *R*_g_ CHROMIXS, PRIMUS σ (nm) | 3.43 ± 0.01 | | 2.87 ± 0.01 | | |
| *min < qR_g_* < *max* limit (or data point range) | 0.16 - 1.21 | | 0.25 – 1.28 | | |
| *PDDF/P(r) analysis* |  | |  | | |
| *I*(0) ± σ (cm^-1;^ a.u.) | 2650 ± 4.22 | | 0.39 ± 0.00 | | |
| *R*_g_  ± σ (nm) | 3.53±0.01 | | 2.89 ± 0.01 | | |
| *d*_max_ (nm) | 13.5 | | 9.1 | | |
| *q*-range (nm^-1^) | 0.05 – 2.33 | | 0.09 – 2.79 | | |
| *P*(*r*) fit assessment | 0.7648 | | 0.9497 | | |
| *Molecular weight (M) estimates (kDa)* |  |  |  |  |  |
| From chemical composition | 66.4 | | 66.4 | | |
| From SAS, concentration-independent method (Bayesian) | 94.2  (84.0 – 110.1) | | 70.6  (68.9 – 105.7) | | |
| From SAS-independent measure (MALS) | na | | 62 kD | | |
| *Shape modelling method(s)* |  | |  | | |
| Software | DAMMIF | | DAMMIF | | |
| *q-*range for fit (*q_min_* – *q_max_*; nm^-1^) | 0.05 – 2.33 | | 0.09 – 2.79 | | |
| Symmetry/anisotropy assumptions | P1 | | P1 | | |
| Number of individual model reconstructions | 10 | | 10 | | |
| *χ*^2^, CorMap *P*-values for fit | 1.087, 0.031 | | 1.015, 0.258 | | |
| *Atomistic modelling methods* |  | |  | | |
| Software | CRYSOL | | CRYSOL | | |
| *q-*range for fit (*q_min_* – *q_max_*; nm^-1^) | 0.03 – 5.00 | | 0.05 – 5.00 | | |
| *χ* ^2^ | 7.521 | | 1.091 | | |
| Data and model deposition |  | |  | | |
| SASBDB IDs | *na.* | | SASDRQ8* | | |

**Supplement Fig. 1: SAXS data processing**


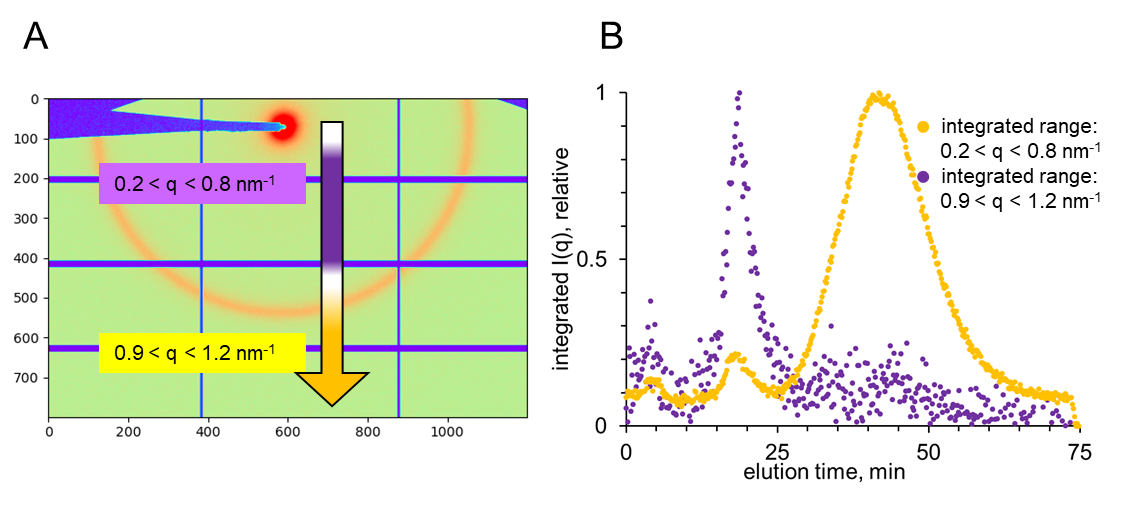


**Supplement Fig. 1 Processing of AF4-SAXS data from lipoplex formulation**

**A.** 2D scattering image from LPX formulation indicating the ranges of intensity used for the integration to generate the SAXS fractionation (elution) profiles. For clarity and pronounced visualization of the characteristic Bragg Peak at 1 nm^-1^, the detector image collected in batch mode at higher concentration is shown.

**B.** SAXS elution profiles from AF4 separation of LPX formulation. The integration at low q highlights the elution of RNA throughout the run (purple). The integration around the Bragg Peak highlights the elution of the lipoplex particles (orange).

**Supplement Fig. 2: LS derived structural information**

**
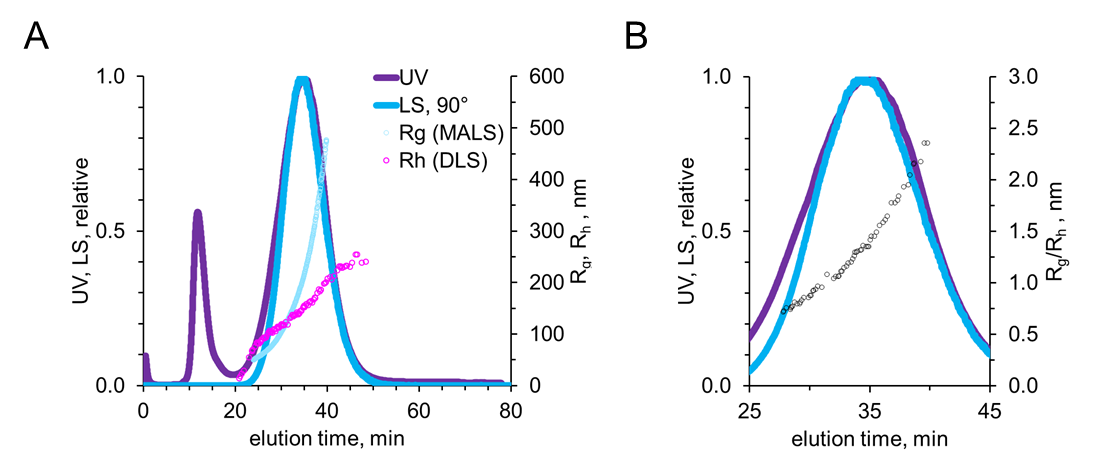
**

**Supplement Fig. 2: Off-line-AF4 measurement**

To confirm that the separation of the particles was not compromised with the increased amount of sample that was loaded (to obtain a stronger scattering signal) an off-line measurement was performed. **A.** The elution profiles are in good agreement with the ones presented in Fig. 3B. In addition to the derived R_g_ values, R_h_ values could also be determined as the dynamic light scattering was measured in addition. **B.** The ratio R_g_/R_h_ is plotted as open spheres. While for a hard sphere a value of ~0.78 is expected, we observed a continuous increase of R_g_/R_h_ up to >2 with larger particle size indicating an increasing deviation of their morphology towards more elongated particles^46^.

**Supplement Fig. 3: SAXS analysis of mRNA peak**


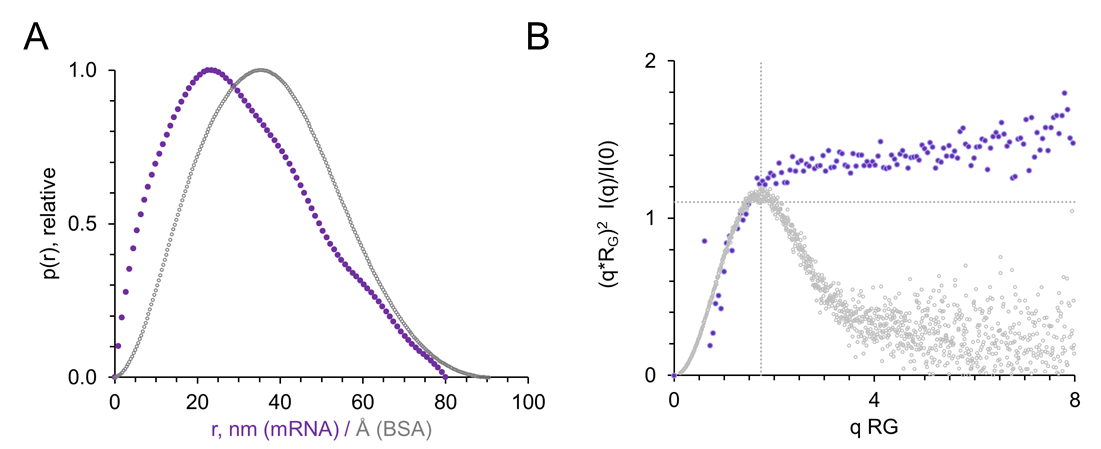


**Supplement Fig. 3: SAXS analysis of free mRNA indicates an unstructured/elongated structure**

The scattering RNA curve from the fractionation of pure RNA formulation was further characterized and shown here in comparison with BSA as an example of a globular structure.

**A.** p(r) function, normalized. The skewed peak is typical for unstructured or elongated structures (in contrast to the almost Gaussian distribution displayed by BSA (grey). Note, the BSA data is displayed in Å for better comparison)

**B.** Dimensionless Kratky plot ((q*R_g_)² I(q)/I(0) vs q*R_g_). The dotted lines are drawn at qR_g_ = √3 and (qR_g_)^2^I(q)/I(0) = 1.104. Globular structures (such as BSA (grey)) have a local maximum where the two lines intersect. The lack of a maximum for the RNA scattering is in agreement with an elongated structure. Concentration-independent MW estimates were in the range of the expected monomer (Bayesian, 440-450 kDa) and in accordance with the MW estimated from MALS.

**Supplement Figure 4: Limitation of SAXS Guinier analysis for larger LPXs**


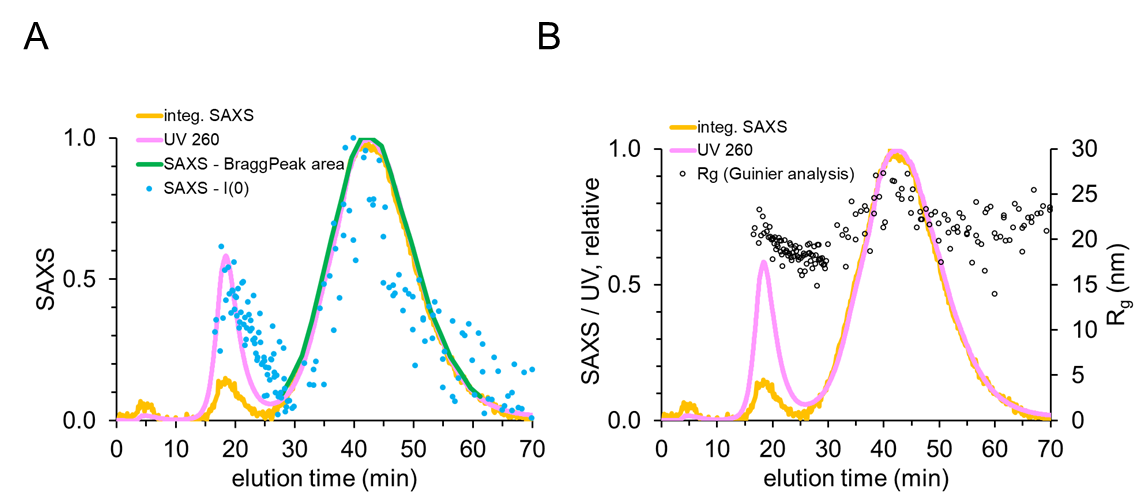


**Supplement Figure 4: Limitation of SAXS Guinier analysis for larger LPX**

A) I(0) and B) R_G_ derived from Guinier analysis (determined with AutoRG) of subtracted frames across the complete fractionation process (both peaks) in comparison with UV (pink) and SAXS trace (orange). Note, due to the overall large size of the nanoparticles with and the limited covered q range for these experiments, the Guinier analysis of the second peak does not reflect the scattering of the overall particle, solely that of the lipid complexed RNA molecules. This explains the constant RG across the peak instead of the continuous size distribution detected with MALS (Figure 1C, black trace). However, the overall good fit of the SAXS elution profile derived from fitting of the Bragg peaks at the various elution time points (green curve) to the various other profiles, justifying its use for the subsequent quantitative data analysis.

**Supplement Fig. 5: extended peak analysis**


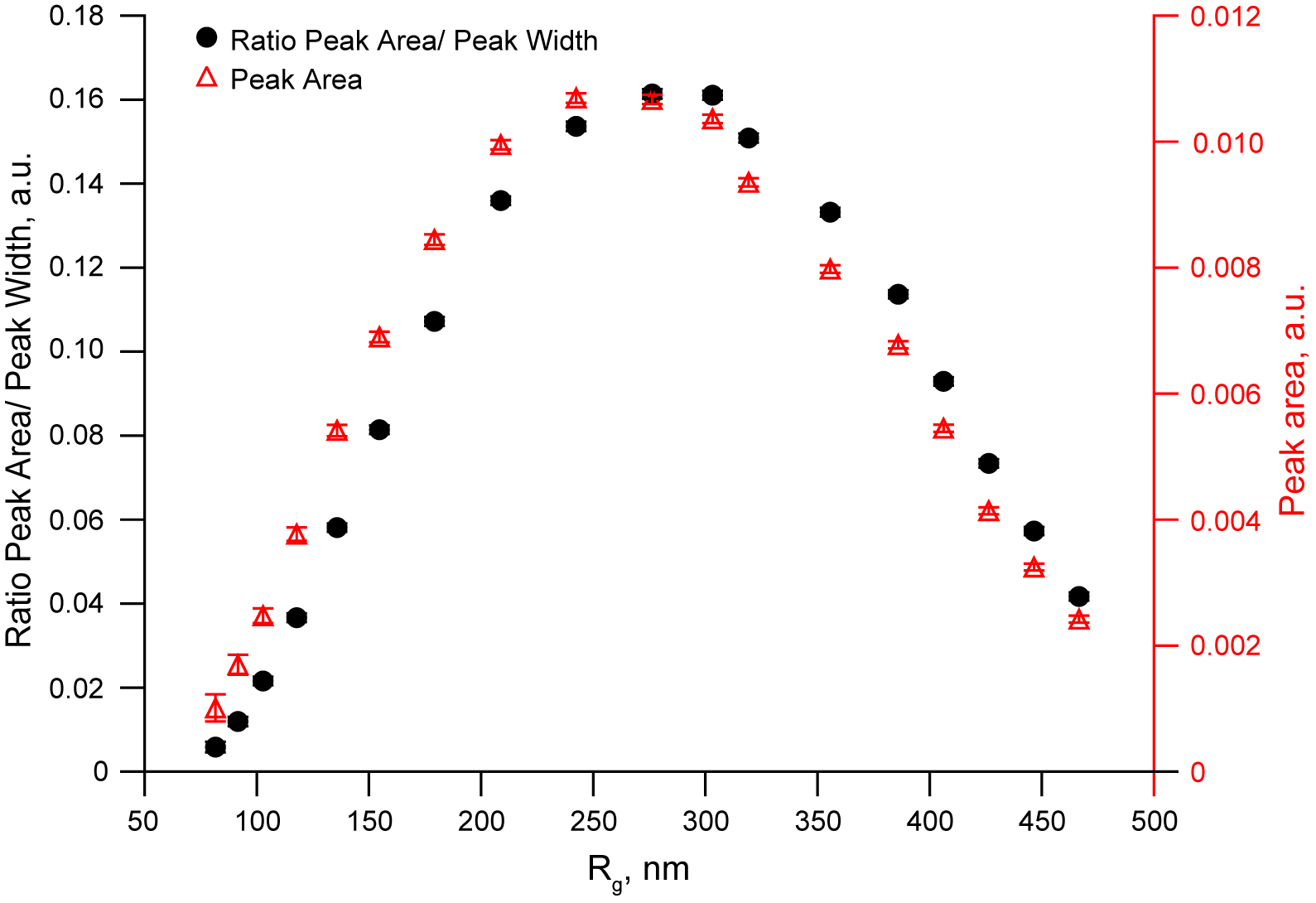


**Supplement Figure 5: Peak area and area/with ratio as a function of R_g_**

Red triangles give the evolution of peak area as a function of R_g_, which shows the same qualitative shape as the plot area vs. elution time (Fig. 4B/D) with a maximum around 250 nm, indicative for the highest fraction of ordered material at this size. Black dots give the ratio peak area/peak width, to highlight an eventual preferred type of organization at a certain size (Papst et al., Structural analysis of weakly ordered membrane stacks, Journal of Applied Crystallography 36(6, 2003, DOI: 10.1107/S0021889803017527). With the rather smooth, continuous change of peak width as a function of particle size/elution time (Fig. 4B) only a slight shift of the maximum towards smaller size is obtained.

**Supplement Table 2:** Peak fitting parameters of lipoplex Bragg peak with Lorentzian function and results of R_g_ analysis of fractions with different elution times

| Elution time  (min) | peak position  (nm^-1^) | σ  (nm^-1^) | peak width  (nm^-1^) | σ  (nm^-1^) | peak area | σ | d-spacing  (nm) | σ  (nm) | Correlation length  (nm) | σ  (nm) | R_g_ |
| --- | --- | --- | --- | --- | --- | --- | --- | --- | --- | --- | --- |
| 29.9 | 1.037 | 5.26E-03 | 0.173 | 2.97E-02 | 1.01E-03 | 2.17E-04 | 6.058 | 3.07E-02 | 11,542 | 1,981 | 81.6 |
| 31.6 | 1.038 | 2.30E-03 | 0.142 | 1.15E-02 | 1.70E-03 | 1.58E-04 | 6.056 | 1.34E-02 | 14,074 | 1,140 | 91.6 |
| 33.2 | 1.038 | 1.13E-03 | 0.115 | 5.07E-03 | 2.47E-03 | 1.16E-04 | 6.050 | 6.57E-03 | 17,436 | 0,771 | 102.8 |
| 34.9 | 1.039 | 6.64E-04 | 0.103 | 2.85E-03 | 3.77E-03 | 1.07E-04 | 6.049 | 3.87E-03 | 19,447 | 0,538 | 117.8 |
| 36.6 | 1.039 | 3.91E-04 | 0.093 | 1.59E-03 | 5.41E-03 | 9.02E-05 | 6.048 | 2.27E-03 | 21,461 | 0,367 | 135.8 |
| 38.2 | 1.039 | 2.80E-04 | 0.085 | 1.10E-03 | 6.90E-03 | 8.47E-05 | 6.048 | 1.63E-03 | 23,599 | 0,306 | 154.7 |
| 39.9 | 1.039 | 2.17E-04 | 0.079 | 8.32E-04 | 8.44E-03 | 8.27E-05 | 6.048 | 1.26E-03 | 25,388 | 0,268 | 179.2 |
| 41.6 | 1.039 | 1.60E-04 | 0.073 | 5.98E-04 | 9.95E-03 | 7.39E-05 | 6.048 | 9.30E-04 | 27,325 | 0,223 | 208.8 |
| 43.2 | 1.039 | 1.49E-04 | 0.070 | 5.50E-04 | 1.07E-02 | 7.59E-05 | 6.049 | 8.71E-04 | 28,738 | 0,227 | 242.4 |
| 44.9 | 1.039 | 1.43E-04 | 0.066 | 5.19E-04 | 1.07E-02 | 7.43E-05 | 6.049 | 8.33E-04 | 30,243 | 0,237 | 276.3 |
| 46.6 | 1.039 | 1.32E-04 | 0.064 | 4.76E-04 | 1.04E-02 | 6.76E-05 | 6.049 | 7.70E-04 | 31,089 | 0,230 | 303.2 |
| 48.2 | 1.039 | 1.41E-04 | 0.062 | 5.03E-04 | 9.35E-03 | 6.64E-05 | 6.049 | 8.21E-04 | 32,260 | 0,262 | 319.3 |
| 49.9 | 1.039 | 1.48E-04 | 0.060 | 5.25E-04 | 7.98E-03 | 6.08E-05 | 6.048 | 8.63E-04 | 33,395 | 0,293 | 355.67 |
| 51.6 | 1.039 | 1.67E-04 | 0.060 | 5.89E-04 | 6.78E-03 | 5.82E-05 | 6.047 | 9.69E-04 | 33,552 | 0,332 | 385.97 |
| 53.2 | 1.039 | 2.03E-04 | 0.059 | 7.15E-04 | 5.45E-03 | 5.75E-05 | 6.046 | 1.18E-03 | 34,102 | 0,415 | 406.17 |
| 54.9 | 1.039 | 2.52E-04 | 0.056 | 8.81E-04 | 4.14E-03 | 5.56E-05 | 6.046 | 1.47E-03 | 35,450 | 0,553 | 426.35 |
| 56.6 | 1.039 | 3.13E-04 | 0.057 | 1.09E-03 | 3.25E-03 | 5.40E-05 | 6.045 | 1.82E-03 | 35,281 | 0,681 | 446.51 |
| 58.2 | 1.039 | 4.42E-04 | 0.058 | 1.55E-03 | 2.42E-03 | 5.62E-05 | 6.045 | 2.57E-03 | 34,467 | 0,924 | 466.59 |
| 59.9 | 1.040 | 5.21E-04 | 0.058 | 1.84E-03 | 1.83E-03 | 5.00E-05 | 6.042 | 3.03E-03 | 34,209 | 1,076 | na |
| 61.6 | 1.040 | 7.73E-04 | 0.055 | 2.69E-03 | 1.24E-03 | 5.18E-05 | 6.041 | 4.49E-03 | 36,143 | 1,758 | na |
| 63.2 | 1.040 | 1.11E-03 | 0.059 | 3.94E-03 | 9.02E-04 | 5.25E-05 | 6.042 | 6.47E-03 | 34,038 | 2,281 | na |
